# Understanding the effect of retirement on health behaviors in China: Causality, heterogeneity and time-varying effect

**DOI:** 10.3389/fpubh.2022.952072

**Published:** 2022-08-15

**Authors:** Ziju Yan, Nan Xiang, Jia Meng, Hang Liang, Zhang Yue

**Affiliations:** School of Public Administration, Zhongnan University of Economics and Laws, Wuhan, China

**Keywords:** retirement, health behavior, anticipatory effect, lagged effect, fuzzy regression discontinuity

## Abstract

Retirement is an important turning point during the course of life, but few studies have examined the effects of retirement on a broad range of health behaviors in China. We use the longitudinal data of the China Health and Nutrition Survey (CHNS) from 2004 to 2015 to conduct empirical analysis. Fuzzy discontinuity regression was used to assess the association between retirement and health behaviors in the entire sample and subgroups based on gender and education. A time-varying effect model was used to measure the anticipatory effect, immediate effect and lag effect of retirement. We observed that the transition to retirement was associated with healthier lifestyle habits, such as reduced smoking and alcohol consumption and increased exercise motivation. However, the transition was associated with worse sedentary behavior. No significant statistical association was found between retirement and sleep duration. Men and those with higher education levels are more likely to experience the impact of retirement. The anticipatory effect suggests that as the statutory pension age is predictable, workers adjust their behaviors 4 and 5 years before retirement. The lagged effect indicates that it takes time to develop new habits; thus, retirees change their behaviors 2–3 years after retirement. The paper discusses possible reasons for our findings and proposes several policy implications from the perspectives of the government and society to facilitate the realization of healthy aging.

## Introduction

The world population is rapidly aging with a greater retirement population and massive societal impacts. China is facing the largest and fastest growth in population aging. By 2020, the proportion of the Chinese elderly population over the age of 60 has reached 18.7% (1). In addition, an increasing number of people are entering retirement, and retired years are steadily extended due to increasing life expectancy. Retirement is an important turning point in an individual's life (2). Dropping out of the labor market is related to changes in available time, economic income, social networks and personal values, all of which are related to one's lifestyle and health outcomes (3). Many studies have found that retirement has an impact on an individual's health (4–7). Although the results have not been consistent, many researchers confirm that health behavior is an important mechanism linking retirement to health (8–10). These studies show that retirement is an important window period of opportunity to reshape one's health behaviors and, more generally, that retirement is a good time for health promotion.

Previous studies have mentioned the association between retirement and health behaviors. Nonetheless, the results have been mixed, with both positive and negative effects on health behavior reported. Several studies have confirmed that people are more likely to quit smoking and drinking, exercise more, or sleep more to adopt a healthy lifestyle after retirement (11, 12). Shrinkage of work-related social networks, increased free time, and peer effects are often used to explain the positive effect (13, 14). Additionally, some researchers found that retirement was associated with less physical activity, more frequent insomnia, and increased smoking and drinking (15–17). These negative results can be explained by the weakening health investment motives and reduced work restraints (13, 14). A few studies have suggested that retirement is related to sedentary behavior (18). Gender differences in the relationship between retirement and health across countries are also of great concern. Retirement may affect men and women differently given their different retirement attitudes and social roles (19), and the effect may be stronger for men (20). Social status can shape one's experience and cognition to promote or prevent healthy habits. For example, one's own education may contribute to increasing knowledge about health behavior–disease links, and economic resources allow one to “buy” better behavior modification programs, medications, or other treatments (21). Both higher levels of education and economic resources decrease the odds of engaging in low physical activity (22). Yet already voices are suggesting that retirement may be more complex than a simple event.

The effect of retirement on health behaviors may not completely coincide with the timing of withdrawal from employment (23). A study in Taiwan suggests that retirement consists of three stages: the near retirement phase, transition period and retirement stability period (24). First, in most countries, the legal retirement age or pension age is fixed and does not change arbitrarily over time. The stability of the retirement system means that the retirement life is predictable. Specifically, workers will have expectations or concerns about retirement life in the preretirement stage and make some preparations and psychological adjustments to successfully transition to retirement life (23). This notion indicates that retirement has a potential anticipatory effect, which has rarely been discussed.

In addition, the retirement effect occurs with a lag. On the one hand, time is required to form a new habit or create conditions that influence health behavior, so the health behavior does not readjust immediately in response to the changes associated with retirement. Evidence in the American population showed that the impact of retirement on cognitive ability has a certain lag effect because it takes time for social networks to shrink (25). On the other hand, smoking and drinking are addictive behaviors that are more difficult to change than other behaviors. Similarly, a recent study based on the French GAZEL Cohort Study (GAZEL stands for *GAZ* and *ELectricité*) found that women had decreased odds of smoking after 5 years compared with 1 year since retirement (26). Although the fuzzy regression discontinuity design is the most widely used method for the topic of retirement, it can only estimate local effects and identify the short-term effects of retirement (27). Liu and Luo (28) found that retirement has a positive short-term effect and a negative long-term effect on cognitive ability (28). Hence, it is necessary to adjust the method to estimate the lag effect and describe the complete picture of retirement.

Contradictory findings of the effect of retirement on health behaviors have been reported. In addition, the role of gender and socioeconomic status has not received sufficient attention, and the importance of the anticipatory effect and lag effect has generally been ignored. Therefore, the present study attempted to address these gaps in several ways. In general, the main purpose of this study is to assess the association between retirement and health behaviors in the entire sample and in subgroups defined by gender and education and to further explore the anticipatory effect and the lag effect of retirement. The marginal contributions of this study mainly include three aspects. First, in contrast most previous studies based on regional and cross-sectional data, we aimed to assess the retirement effect on health behaviors through a nationwide population-based study with long-term follow-up data, which could lead to more robust and comprehensive results. Second, differences in gender and SES were further analyzed in the present research. Third, based on the statutory retirement system in China, we took the retirement stage into consideration to estimate the anticipatory effect and the lag effect, as these topics have not received much attention in the literature. Based on the above analysis, we raise concerns for the statutory retirement system and propose corresponding policy suggestions for health intervention to improve the health behavior and health outcomes of the retiring and retired elderly.

## Data and methodology

### Data source and study population

This study draws on data from the China Health and Nutrition Survey (CHNS), an international collaborative project between the Carolina Population Center at the University of North Carolina at Chapel Hill and the National Institute for Nutrition and Health at the Chinese Center for Disease Control and Prevention. The CHNS is a panel dataset that has collected data every 2–4 years since 1989 followed by nine waves of surveys in 1991, 1993, 1997, 2000, 2004, 2006, 2009, 2011, and 2015. The stratified multi-stage cluster sampling method was used to recruit CHNS participants from 216 communities in China with a response rate of 88% at individual level and 90% at household level. Only pre-schoolers and young adults aged 20–45 years were surveyed in 1989 due to constraints of funding. Since 1993, in each wave of the CHNS survey, the sample has been composed of the households originally sampled in 1989 plus all new households formed from sample households who resided in sample areas. More details about the sampling can be found elsewhere (29). CHNS uses a wide range of indicators, including income, employment, health and nutrition, demographic variables of respondents. Such a wide range of indicators enables an estimation of retirement effect.

**Figure 1:**
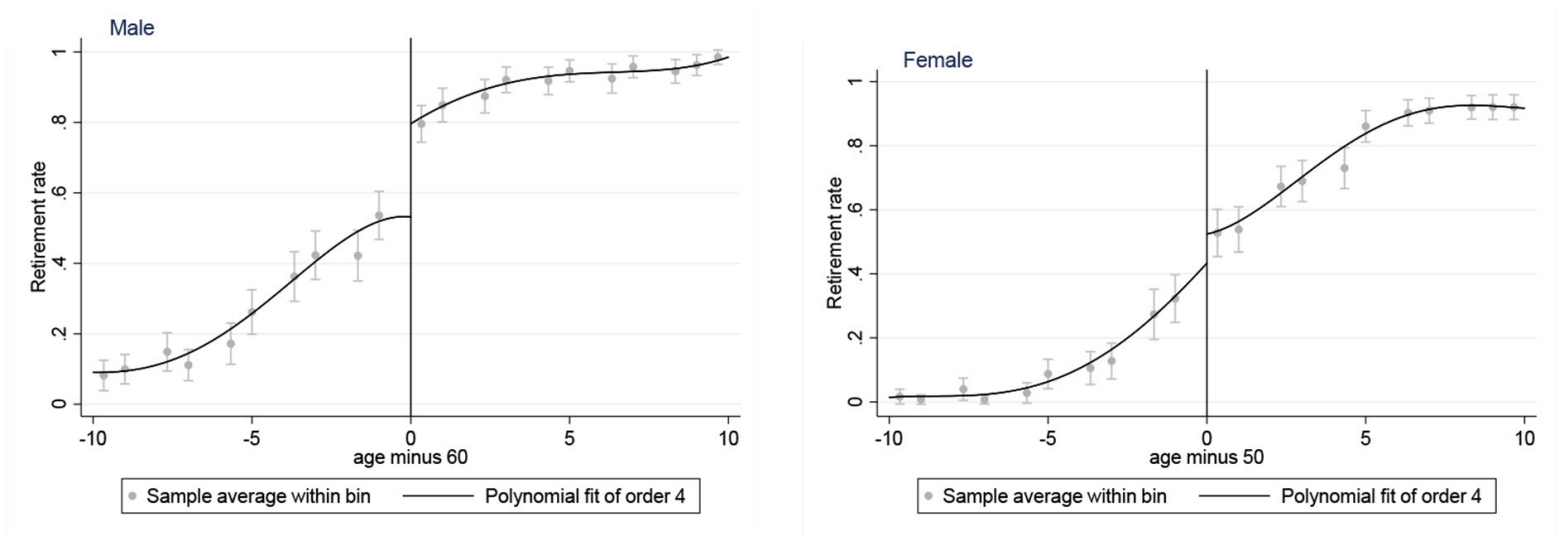
Retirement rate by age among males and females. The vertical lines at ages 50 and 60 are the statutory retirement ages for females and males.

We only included the 2004–2015 CHNS surveys in the analysis because the first five surveys lack some key indicators, such as sleep duration and smoking history. First, we restrict the retirement group to individuals who are not working due to retirement to exclude interfering factors, such as the temporary unemployment. This reduces the observations from 100,821 to 83,476; Second, given the government departments, public institutions, state-owned enterprises, and collectively owned enterprises can more strictly implement the statutory retirement system in China (30), we further excluded samples not in these units (30). The sample size is reduced to 59,787. Third, we use an age range of 10 years to control the age effect (31). The statutory retirement age in China is 60 years for men, 50 years for most female workers (see Figure 1). Thus, we restrict our observations to men aged 50–70 and women aged 40–60 in each wave. This reduces the observations from 59,787 to 7,799; Lastly, listwise deletion procedures are used to handle the missing data. As a result, in total, the sample size in these five waves accumulated to 7,330 with 4,190 participants.

Among the 4,190 respondents, 31% (1,288) of the respondents only contributed the pre-retirement data; 29% (1,218) have contributed both pre-retirement data and post-retirement data; 40% (1,684) only contributed the post-retirement data. On average, each respondent was observed twice. Specifically, 1,862 (44%) were observed only once; 1,267 (30%) were observed twice; 525 (13%) were observed three times, 396 (9%) were observed four times; only 140 (3%) were observed five times.

Our sample is 52% men and 69% experienced the retirement event during the observation period. The mean age of male sample is 58 years with a range of 50 to 70 years, and the mean age of female sample is 49 years with a range of 40 to 60 years. Ninety-three percent of the sample are married with a couple and 24% have at least one chronic disease. Most of them have completed China's 9-year compulsory education. Sixteen percent of the sample just finish the primary school and 17% have college or higher education.

On average, there was an 18% of attrition rate due to refusal, above the age threshold, difficulties to relocate as of migration or urban resettlement. The rate was similar to those internationally well-known surveys (32). Without a 100% rate of completion of the survey, there is always a possibility of selection bias (33). Those who completed the survey may be different from those who did not take the survey. Thus, we compared our sample to previous studies with different data (8, 9). Major demographic characteristics of the sample data were very similar.

### Variables

#### Explained variables: Health behaviors

The study chose smoking, drinking, physical exercise, sleeping and sedentary behavior as dependent variables (22). Because they are closely related to people's health and show beneficial or detrimental to people's health (2–5). Each behavior was measured as follows:

The smoking and drinking variables were measured based on the total number of cigarettes smoked per day and the number of alcoholic beverages consumed per week reported by respondents. Given that a study reported that retirement only affected participants who had a history of smoking and drinking, smoking and drinking behaviors were initially restricted to these groups (34). In addition, participants without smoking and drinking histories were introduced into the follow-up robustness test to analyse the effect of retirement on the probability of individual smoking and drinking.

Exercise refers to the individual's preference for participating in various physical activities, such as walking, tai chi, table tennis, badminton and indoor fitness. The questionnaire assessed preference based on a score ranging from 1 (not like at all) to 5 (like it very much). We further aggregated the scores. The higher the score, the greater the motivation to participate in the exercise. The survey highlights the motivation of individuals to participate in sports rather than watch as a spectator, so this motivation instrument has been found to have acceptable reliability and validity (35). In the following robustness check, we also take into account the kinds of activities in which the participants participate.

Sitting behavior and sleep were measured by asking respondents how many hours each 24-h weekday they usually spend watching TV and sleeping. Sedentary behavior refers to activities that do not consume significant energy, including sitting, watching television, and other screen-based entertainment (36). For middle-aged and elderly people, the main sedentary activity is watching TV, while for young people, it's using the tablet or smartphone. Thus, the sedentary behavior is typically measured by the time spent watching TV (37). Considering that retirement will directly affect the schedule of the workday, sedentary behavior is measured by the average amount of time spent watching TV during the workday.

#### Explanatory variable: Retirement status

The key independent variable is the retirement status of individuals, which is defined as a person who is not working and the reason for not working is retirement. Two questions are used to assess retirement status. First, the respondent is asked “Are you presently working?”. Then, those who answer “no” are asked “Why are you not working?”. The answers to this question include “seeking work”, “doing housework”, “disabled”, “retired”, “other” and “unknown.” Those who answer “retired” are retained for the analysis; otherwise, the respondents are excluded. As a result, our retirement status variable equals 1 if the respondent is retired and 0 if he or she is still working.

#### Control variables

First, age and the polynomial of age were considered given that Figure 1 shows that the retirement rate rises with age. Controlling age and its polynomial can control the effect of age and construct a non-linear relationship to avoid estimation bias caused by the correlation between age and random disturbance terms. In addition, there are several predetermined variables, including gender (male coded as 1), marital status (married coded as 1), chronic disease (yes coded as 1), years of education and province (yes coded as 1). All of the above predetermined variables are binary variables except for years of education.

### Model settings

#### Establishing the fuzzy regression discontinuity design model

Endogenous problems exist in the impact of retirement on residents' health behavior. (i) Individual retirement preference, family health endowment, subjective life expectancy and other unobservable missing variables can cause evaluation bias and (ii) the problem of reverse causality between retirement and health behaviors.

To address the endogenous problem, this paper draws on Lee and Lemieux to employ the fuzzy regression discontinuity design (FRD) method (38). Specifically, using statutory retirement age policies as instrumental variables for participants' retirement status, the effect of retirement is estimated. Hence, the equations for the relationship among actual retirement status, statutory retirement age system, and health behaviors are constructed as follows.


(1) $$T_{i}=\;\{\begin{array}{c} 0,\;if\;M\;<0\\ 1,\;if\;M\;\geq 0 \end{array}$$

(2) $$\begin{array}{l} D_{i,t}\;=\;\alpha_{0}\;+\;\alpha_{1}T_{i,t}\;+\;\alpha_{2}X_{i,t}\;+\;f\left(M\right)\\ \;+\mu_{i,t}\;+\;\delta_{i,t}\;+\;\varepsilon_{i,t} \end{array}$$

(3) $$\begin{array}{l} Y_{i,t}\;=\;\beta_{0}+\beta_{1}D_{i,t}\;+\;\beta_{2}\;X_{i,t}\;+\;f(M)\\ \;+\;\mu_{i,t}\;+\;\delta_{i,t}\;+\;\varepsilon_{i,t} \end{array}$$

M equals the difference between the actual age of the individual and the statutory retirement age. If the individual has reached the legal retirement age, the instrumental variable *T*_*i*_ equals 1; otherwise, it equals 0. Then, we construct Equation (2) for the instrumental variables of the statutory retirement age and actual retirement behavior. Finally, the fitted values of *D*_*i,t*_ obtained from Equation (2) are substituted into Equation (3) to estimate the effect of retirement on various health behaviors.

*Y*_*i,t*_ refers to the various health behaviors of individual i in year t, and *D*_*i,t*_ denotes the retirement status of individual i in year t. *T*_*i,t*_ are the instrumental variables. *X*_*i,t*_ is a vector of covariates that affect health behaviors, and *f*(*M*) are polynomials of the participants' age. ε_*i,t*_ is a random disturbance term, and μ_*i,t*_ and δ_*i,t*_ are the year fixed effect and the province fixed effect, respectively.

#### Establishing the time-varying effect model

The previous analysis implicitly assumes that transitioning to retirement results in an immediate change in an individual's health behaviors, and the change is persistent and constant. As long as the individual switches from working to retirement, the health behavior changes correspondingly. However, the treatment effects of retirement are not constant over time. It is therefore important to allow for time varying treatment effects when estimating panel data models (39), and both the anticipatory and lagged effects should be considered.

To effectively measure the anticipatory and lagged effects of retirement, we set the following time-varying effect model by introducing pulse variables, which is proposed by Laporte and Windmeijer (39):


(4) $$\begin{array}{l} Y_{it}\;=\;\beta_{0}\;+\;\beta_{1}X_{i,t}\;+\;\ldots \;+\;\gamma_{-2}P_{i,-2}\;+\;\gamma_{-1}P_{i,-1}\;+\;\gamma D_{i,t}\\ \;+\;\gamma_{0}P_{i,0}\;+\;\gamma_{1}P_{i,1}\;+\;\gamma_{2}P_{i,2}\;+\;\ldots \;+\;\varepsilon_{i,t} \end{array}$$

where *P*_*i, j*_ is the indicator variable and j=-2,−1, 0, 1, and 2, which represents j periods after (before) the introduction of the treatment, namely, retirement. The indicator variable is 1 in the j-th period after (before) retirement; otherwise, it is 0. According to the CHNS tracking period, when *P*_*i*, ±2_ =1, the participant is in the period of 4–5 years after (before) retirement. When *P*_*i*, ±1_ =1, the individual is in the period of 2–3 years after (before) retirement. When *P*_*i*, 0_ =1, the individual is in the year of his retirement or 1 year after retirement.

Using this method, we were able to estimate the anticipatory effect—γ_−1_ and γ_−2_ of retirement on health behavior, immediate effect—γ_0_ and lagged effect—γ_1_ and γ_2_. The remaining variables have the same meaning as noted in Equation (3).

## Results

### Basic descriptive analysis

Table 1 shows the measurement and descriptive statistics of each variable in this study. As noted in Table 1, it is difficult to determine whether the retiree has a healthier lifestyle. Although retirees seem to smoke less than workers, the retirees experience more sitting time than workers. In addition, minimal differences in drinking, sleeping and exercising are noted between retirees and workers.

**Table 1:** Descriptive statistics.

**Variables**	**Total (7,330)**		**Working (3,182)**		**Retired (4,148)**	
	**Mean**	**S.D**.	**Mean**	**S.D**.	**Mean**	**S.D**.
Smoking	12.923	10.509	15.509	10.585	11.109	10.074
Drinking	8.335	10.511	8.091	10.067	8.546	10.730
Exercise	8.095	2.519	8.097	2.579	8.096	2.468
Sitting	148.769	116.147	131.694	110.968	161.706	118.311
Sleep	7.700	1.141	7.703	1.011	7.696	1.231
Age	55.683	7.314	50.763	6.073	59.439	5.794
Chronic disease	0.252	0.434	0.172	0.378	0.313	0.464
Marriage	0.935	0.247	0.940	0.238	0.931	0.253
Education	10.532	3.814	11.619	3.438	9.704	3.878
Gender	1.474	0.499	1.521	0.500	1.438	0.496

Data were pooled the observations of the CHNS 2004-2015.

### Results of fuzzy regression discontinuity design

Figure 1 shows the relationship between the retirement rate and standardized age (actual age minus legal retirement age). Figure 1 shows that at the legal retirement age, both male and female retirement rates increase significantly.

Table 2 further reports the results of the first stage of the FRD to estimate the association between retirement behavior and the statutory retirement age system in all participants and different gender groups after adjusting for the covariates based on longitudinal data from 2004 to 2015. In Columns 1–3, the legal retirement system was significantly and positively related to the rate of retirement after adjusting for the covariates, which is consistent with the results reported in Figure 1.

**Table 2:** Regression results from the first stage of the FRD.

**Variables**	**Retired**		
	**Male**	**Female**	**Total**
Retirement system	0.294*** (0.019)	0.241*** (0.022)	0.263*** (0.015)
Standardized age	0.033*** (0.002)	0.038*** (0.002)	0.036*** (0.001)
(Standardized age)^2^	−0.002*** (0.000)	0.000** (0.000)	−0.001*** (0.000)
Control variables	YES	YES	YES
Year fixed effects	YES	YES	YES
Province fixed effects	YES	YES	YES
Observation	3,846	3,484	7,330
Wald chi^2^	3297.05***	3050.79***	6183.26***
*R* ^2^	0.561	0.567	0.557

**p < 0.05, ^***^p < 0.01. Standard errors are reported in parentheses.

Table 3 presents the estimates and the standard errors of the FRD model to explore the association between retirement status and health behaviors in all participants. Both smoking and drinking behaviors were reduced. The result shows that the effect of retirement on smoking behavior is −6.548 (*p* < 0.05), and the effect of retirement on drinking behavior is −7.838 (*p* < 0.05). Retired subjects had a greater preference for practicing physical activities than subjects who did not exit the workforce (B = 1.01, *p* < 0.01), but transitioning to retirement led to a 37-min increase in the time of sedentary behavior (B = 37.11, *p* < 0.1). Sleep duration did not change between the exposed and nonexposed subjects (B = 0.074, *p* > 0.1).

**Table 3:** Regression results from the second stage of the FRD.

**Variables**	**Health behaviors**				
	**Smoking**	**Drinking**	**Exercise**	**Sleep**	**Sitting**
Retired	−6.548**	−7.838**	1.010***	0.074	37.110*
	(2.689)	(3.356)	(0.379)	(0.189)	(19.656)
Standardized age	−0.025	0.384**	−0.032	−0.012	−0.4033
	(0.153)	(0.191)	(0.021)	(0.011)	(1.106)
(Standardized age)^2^	−0.015**	0.004	0.002***	0.001	−0.058
	(0.007)	(0.008)	(0.001)	(0.000)	(0.045)
Control variables	YES	YES	YES	YES	YES
Year fixed effects	YES	YES	YES	YES	YES
Province fixed effects	YES	YES	YES	YES	YES
Observation	2,251	1,837	7,330	7,330	7,330
Wald chi^2^	171.910***	194.410***	2414.032***	165.046***	222.300***
*R* ^2^	0.087	0.115	0.293	0.036	0.036

*p < 0.1, ^**^p < 0.05, ^***^p < 0.01. Standard errors are reported in parentheses.

Combined with the above analysis, this study reveals a significant positive effect of retirement on individual smoking, drinking and physical exercise behavior, but retirement also has a negative impact on sedentary behavior.

### Results of heterogeneity by gender

Table 4 shows the association between retirement and health behavior in different gender groups. The results stratified by gender indicated a gender difference in the relationship between retirement and health behavior. Retirement significantly reduced males' smoking (B = −5.843, *p* < 0.05) and drinking (B = −8.052, *p* < 0.05) and improved exercise behavior (B = 0.946, *p* < 0.05), whereas it also increased their sitting time (B = 40.568, *p* < 0.1). However, the relationship was not significant in women except for physical exercise (B = 1.064, *p* < 0.1). This finding suggested that men were more likely to be affected by retirement events.

**Table 4:** Heterogeneity by gender.

**Variables**	**Health behaviors**				
	**Smoking**	**Drinking**	**Exercise**	**Sitting**	**Sleep**
**Panel A: Male**					
Retired	−5.843**	−8.052**	0.946**	40.568*	0.001
	(2.426)	(3.264)	(0.460)	(24.342)	(0.237)
Age polynomial	YES	YES	YES	YES	YES
Control variables	YES	YES	YES	YES	YES
Year/Province fixed effects	YES	YES	YES	YES	YES
Observations	2,183	1,643	3,846	3,846	3,846
*R* ^2^	0.089	0.100	0.287	0.040	0.038
**Panel B: Female**					
Retired	−18.383	1.389	1.064*	30.584	0.076
	(24.603)	(5.110)	(0.621)	(30.843)	(0.296)
Age polynomial	YES	YES	YES	YES	YES
Control variables	YES	YES	YES	YES	YES
Year/Province fixed effects	YES	YES	YES	YES	YES
Observations	68	194	3,484	3,484	3,484
*R* ^2^	0.362	0.149	0.306	0.035	0.056

*p < 0.1, ^**^p < 0.05. Standard errors are reported in parentheses.

### Results of the heterogeneity by education

The results stratified by education level are shown in Table 5. For the group with an education level greater than junior high school, both smoking (B = −9.595, *p* < 0.01) and physical activity (B = 1.143, *p* < 0.05) were significantly changed after transitioning to retirement life. However, for those with less than a middle school education, only drinking behavior significantly decreased after retirement (B = −14.551, *p* < 0.05). An increase in sedentary behavior was also noted in these individuals (B = 58.65, *p* < 0.1). Health behaviors are more likely to be improved in groups with higher levels of education.

**Table 5:** Heterogeneity by education.

**Variables**	**Health behaviors**				
	**Smoking**	**Drinking**	**Exercise**	**Sitting**	**Sleep**
**Panel C: < 9 years of education (education≤9)**					
Retired	−3.716	−14.551**	0.899	58.650*	−0.038
	(4.830)	(7.178)	(0.701)	(35.174)	(0.389)
Age polynomial	YES	YES	YES	YES	YES
Control variables	YES	YES	YES	YES	YES
Year/Province fixed effects	YES	YES	YES	YES	YES
Observation	1,189	849	3,313	3,313	3,313
*R* ^2^	0.108	0.143	0.304	0.039	0.041
**Panel C: More than 9 years of education (education>9)**					
Retired	−9.595***	−5.392	1.143**	19.584	0.175
	(3.605)	(3.417)	(0.486)	(25.700)	(0.225)
Age polynomial	YES	YES	YES	YES	YES
Control variables	YES	YES	YES	YES	YES
Year/Province fixed effects	YES	YES	YES	YES	YES
Observation	1,062	988	4,017	4,017	4,017
*R* ^2^	0.090	0.118	0.203	0.045	0.032

*p < 0.1, ^**^p < 0.05, ^***^p < 0.01. Standard errors are reported in parentheses.

### Specification test

**Figure 2:**
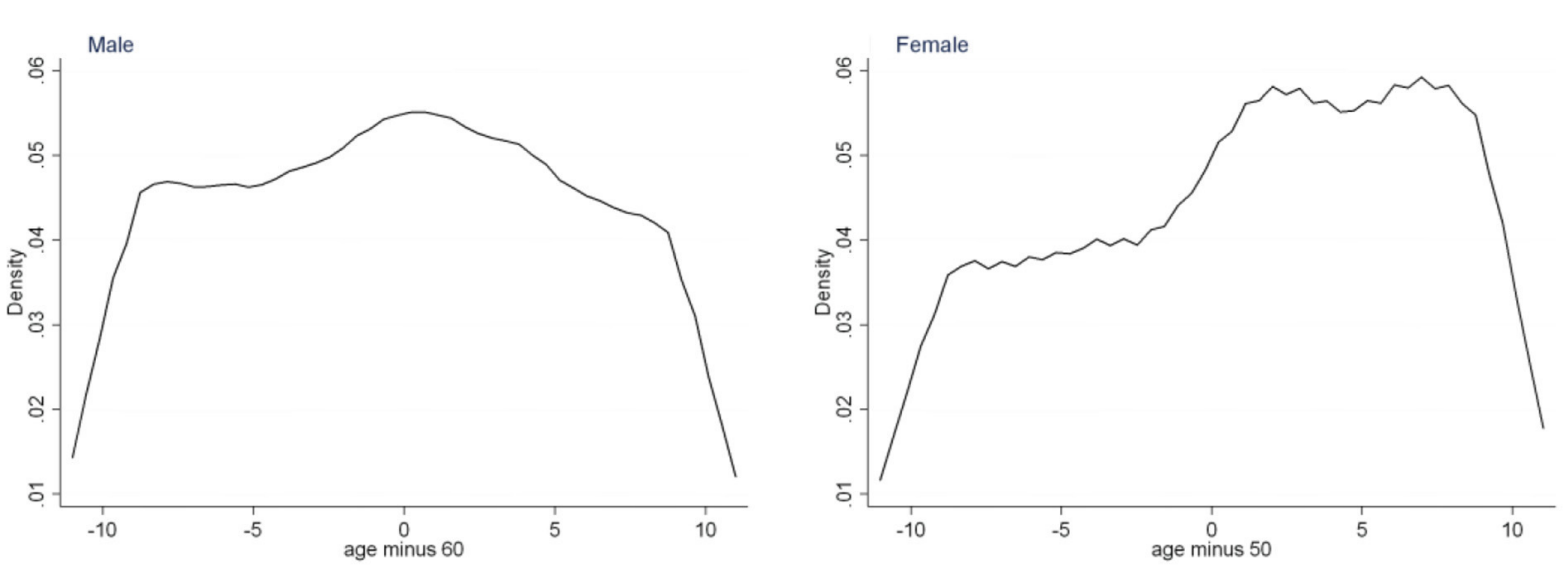
Density distribution of age.

The validity of FRD depends on two assumptions. (1) The running variable (age) cannot be manipulated by respondents, or the effectiveness of the experimental group and the control group cannot be guaranteed. In view of this, we examine the density distribution of age. Figure 2 shows that the age density distribution of males and females is continuous and smooth at their age cut-off point, which can satisfy the assumption that the running variables cannot be manipulated.

(2) The predetermined variables will not “jump” at the cut-off points, or it is difficult to determine whether the “jump” in the health behavior is caused by the running variable or the predetermined variable. In view of this, we examine the continuity of the predetermined variables, which is shown in Table 6. As we can see, none of the estimates are significant; that is, predetermined variables continue at the cut-off point, thus satisfying assumption (2).

**Table 6:** Continuity test of predetermined variables.

**Variables**	**Predetermined variables**		
	**Chronic disease**	**Education level**	**Marriage**
Retired	0.008 (0.016)	0.108 (0.094)	0.011 (0.007)
Age polynomial	YES	YES	YES
Control variables	YES	YES	YES
Year/Province fixed effects	YES	YES	YES
Observation	7,330	7,330	7,330

Standard errors are reported in parentheses.

### Robustness check

#### Use of other dependent variables

For robustness checks, we use two dummy variables of whether the participants smoked and drank to measure smoking and drinking behavior (yes coded as 1). Exercise behavior was measured by the number of activities involved, and sedentary behavior was measured by reading time during the workday. Sleep is also a binary variable, with < 7 h/day or more than 8 h/day being coded as “0” and 7–8 h/day being coded as “1” based on empirical evidence of sleep and health outcomes (18). We replaced the original health behavior variables for regression. The results are shown in Table 7. Except for drinking behavior, the other health behaviors are basically consistent with the results in Table 3 above, so the results of this paper are robust.

**Table 7:** Results of using other dependent variables.

**Variables**	**Health behaviors**				
	**Smoking**	**Drinking**	**Exercise**	**Sleep**	**Sitting**
Retired	−1.838*	0.216	0.149*	−0.635	35.300**
	(1.037)	(0.608)	(0.081)	(0.432)	(16.777)
Age polynomial	YES	YES	YES	YES	YES
Control variables	YES	YES	YES	YES	YES
Year fixed effects	YES	YES	YES	YES	YES
Province fixed effects	YES	YES	YES	YES	YES
Observation	7,330	7,330	7,330	7,330	3,440

*p < 0.1, ^**^p < 0.05. Standard errors are reported in parentheses.

#### Bandwidth sensitivity test

In the sensitivity test of bandwidth, we restrict the bandwidth from the original [−10, 10] to [−3, 3] and [−8, 8]. The results are shown in Tables 8, 9. The results were basically consistent with Table 3 regardless of the variation in bandwidth. Hence, the conclusions obtained in this paper are robust.

**Table 8:** Sensitivity test of window width 3.

**Variables**	**Window width** = **3**				
	**Smoking**	**Drinking**	**Exercise**	**Sleep**	**Sitting**
Retired	−17.732**	−15.544*	0.502	0.915*	108.234**
	(7.211)	(8.739)	(0.970)	(0.501)	(53.359)
Age polynomial	YES	YES	YES	YES	YES
Control variables	YES	YES	YES	YES	YES
Year fixed effects	YES	YES	YES	YES	YES
Province fixed effects	YES	YES	YES	YES	YES
Observation	839	682	2,632	2,632	2,632

*p < 0.1, ^**^p < 0.05. Standard errors are reported in parentheses.

**Table 9:** Sensitivity test of window width 8.

**Variables**	**Window width** = **8**				
	**Smoking**	**Drinking**	**Exercise**	**Sleep**	**Sitting**
Retired	−8.609**	−12.534***	1.005**	0.224	34.575
	(3.442)	(4.278)	(0.489)	(0.243)	(25.311)
Age polynomial	YES	YES	YES	YES	YES
Control variables	YES	YES	YES	YES	YES
Year fixed effects	YES	YES	YES	YES	YES
Province fixed effects	YES	YES	YES	YES	YES
Observation	1,876	1,521	6,067	6,067	6,067

**p < 0.05, ^***^p < 0.01. Standard errors are reported in parentheses.

#### Results of the time-varying model

In this section, a time-varying effect model was used to estimate three possible effects of retirement. To determine the retirement period an individual was in, the samples that had retired in the first wave of being interviewed and those who had only been interviewed once were excluded. Given that we did not find a significant relationship between sleep duration and retirement, it was excluded from this section. The results are shown in Table 10.

**Table 10:** Results of the time-varying model.

**Variables**	**Health behaviors**			
	**Smoking**	**Drinking**	**Exercise**	**Sitting**
P_−2_	0.545 (0.902)	−2.749*** (0.937)	0.042 (0.117)	11.432** (5.221)
P_−1_	0.558 (0.816)	−1.659* (0.902)	−0.017 (0.107)	2.631 (4.919)
P_0_	−1.246 (0.858)	−1.412 (0.930)	0.099 (0.107)	25.794*** (4.920)
P_1_	−2.488** (1.063)	−0.313 (1.236)	0.283** (0.142)	22.923*** (6.681)
P_2_	−2.386* (1.322)	−2.454 (1.514)	0.174 (0.168)	21.287*** (7.733)
Retired	−4.954	−4.123	−0.39	32.603
	(3.444)	(5.162)	(0.485)	(24.380)
Age polynomial	YES	YES	YES	YES
Control variables	YES	YES	YES	YES
Year fixed effects	YES	YES	YES	YES
Province fixed effects	YES	YES	YES	YES
Observation	2,102	1,742	6,127	6,127

*p < 0.1, ^**^p < 0.05, ^***^p < 0.01. Standard errors are reported in parentheses.

#### The anticipatory effect

The results show that the anticipatory effect of retirement is mainly reflected in drinking behavior and sedentary behavior. Drinking behavior decreased significantly 4–5 years before retirement (B = −2.749, *p* < 0.01), but it was also accompanied by an increase in sedentary behavior (B = 11.432, *p* < 0.05). No significant change in smoking or exercise behavior was noted before retirement.

#### The immediate effect

The results of the immediate effect in Table 10 show that the immediate effect of retirement only existed in sitting behavior with an immediate and significant increase in leisure sitting time as retirement status shifted (B = 25.794, *p* < 0.01). No significant changes in other health behaviors that required time to change were noted.

#### The lagged effect

Regarding the lagged effect, we can see that smoking, exercising, and sedentary behaviors change significantly after 2–3 years of retirement. Specifically, smoking behaviors are significantly reduced (B = −2.488, *p* < 0.05), and the preference for exercise significantly improves (B = 0.283, *p* < 0.05). However, sedentary behaviors also increase (B = 22.923, *p* < 0.01). Among these behaviors, smoking (B = −2.386, *p* < 0.1) and sedentary behaviors (B = 21.287, *p* < 0.01) continued to change at 4–5 years after retirement. The lag effect of retirement was very significant.

## Discussion

This large population-based study in China found that retirement was associated with an improvement in health behaviors characterized by less smoking and drinking and a stronger preference for exercise. However, retirement also increased sedentary behaviors. In addition, we did not obtain significant evidence about the relationship between retirement and sleep duration. As one of the first studies to comprehensively examine a broad range of lifestyle behaviors in China, the current study implies that retirement could represent a positive transition to a healthier lifestyle.

In this study, significantly reduced smoking was noted in individuals after retirement, which is consistent with that reported in most studies. Kesavayuth et al. (40) analyzed 10 European countries based on the instrumental variable method and found that residents with a smoking history significantly reduced their smoking after retirement, which could be explained by peer effects (40). However, our heterogeneity analysis showed that the positive effect of retirement on smoking behavior only existed in the male group. Retirement did not significantly affect smoking behaviors in women. This finding differed from the results from a study in France that reported that women, but not men, were more likely to quit smoking after retirement (26). Cultural context can account for these differences (40), as strong taboos against female smoking exist in much of Asia (41), which leads to a small smoking group among Chinese women. Socioeconomic status is closely related to one's health behavior (42, 43). Education, occupation, and income are typically used to measure socioeconomic status, but occupation and income change as an individual enters retirement. Thus, we use education level to reflect socioeconomic status (44). It can be seen that the positive effect of retirement on smoking behavior mainly exists in the group who completed junior high school. For people of lower socioeconomic status, retirement means less income from work. Individuals who lack financial savings will experience financial stress, which is not conducive to the development of health behaviors (45).

Our data revealed that retirees reduced alcohol consumption, echoing results from some previous studies (46). Considerable research on retirement and alcohol consumption has been performed. Wang et al. (47) found that older adults may drink more frequently after retirement due to reduced work restrictions and increased leisure time (47). However, Zou et al. (31) found that retirement weakens work-related social networks and reduces the probability of drinking (30). Some research even found that a significant relationship does not exist between retirement and alcohol consumption (48). We further tested the gender differences in the effect of retirement on drinking. Retired men reported less alcohol consumption than men not yet retired, but there was no significant difference between retired and working women, which supports previous studies (47, 49). It is possible that small sample sizes prevented inferences about women because, generally, there are more drinkers among men and men are able to biologically tolerate more alcohol than women (50). Richman et al. (51) noted that the degree of work stress is an important factor in reversing this relationship (51). In addition, we also found that this reduction occurred mainly among those who did not complete compulsory education. A possible explanation for this discrepancy is that individuals with higher socioeconomic status generally have more opportunity to engage in social activities that are often associated with alcohol consumption (52). The disappointment paradox theory also suggests that loss of job-related status may be worse for higher socioeconomic groups because they are less used to coping with retirement (53, 54).

In the present study, retirees reported a significantly greater increase in motivation to participate in activities. The principal reasons for this finding are the increase in leisure time and the decrease in work stress. Further analysis found that the positive effect of retirement on exercise was reflected in both men and women, indicating that retirement is indeed an important window for improving exercise behavior (35). However, the analysis of educational heterogeneity showed that there was no significant difference in sports participation between residents of lower socioeconomic status before and after retirement. On the one hand, people with low socioeconomic status are more inclined to engage in physical labor during work, and it is difficult for residents to increase their enthusiasm for physical exercise after retirement. On the other hand, for people of lower socioeconomic status, financial pressures from their families may motivate them to engage in gardening and household chores, which also decrease their motivation to exercise (12).

Consistent with earlier studies using self-reported sedentary time primarily based on leisure time activities or exclusively on TV viewing (55, 56), residents experienced a marked increase in sedentary time after retirement, which may be explained by increased amount of free time at home. Further analysis of gender heterogeneity found that the average sedentary time after retirement increased by 41 min for men but not significantly for women. Women are more likely to be influenced by positive social support and neighborhood social cohesion, and men appear to be less influenced by these forces (57). Additionally, women do housework more frequently than men, especially at this age (58). The less-educated group experienced a 59-min increase in sedentary time after retirement, whereas sedentary time did not increase significantly in the more-educated group. Suorsa et al. (59) used occupational status to reflect participants' socioeconomic status, and they argued that people with higher SES tend to engage in less sedentary behavior after retirement because they consciously remain active compared to people with lower SES (59).

Finally, sleep duration was not associated with retirement in the current study, and the findings remained robust after sensitivity analyses. A recent review on retirement and sleep found that although an increase in leisure time will theoretically increase one's sleep time, some studies have shown that the elimination of work stress after retirement can significantly improve an individual's sleep disorders and sleep quality; thus, the sleep duration is less important (60).

The most important and surprising contribution of this study was the investigation of the anticipatory effect, the immediate effect and the lagged effect of the retirement system. The anticipatory effect refers to individuals' health behaviors changing 4–5 years before retirement, which are mainly reflected in drinking and sedentary behavior. Social entertainment at work is usually accompanied by drinking. Approaching the retirement period, work affairs gradually decreased, and residents' drinking behavior decreased significantly. Vigezzi et al. (23) mentioned that “the statutory pension age is predictable, workers may adjust their behaviors before retirement” as their limitation (23). However, these authors did not provide further answer for this limitation. Given the lack of significant difference in smoking between working and retired Korean men, Kim et al. (61) surmised that smoking and alcohol cessation programs had been implemented during employment (61). The immediate effect is mainly reflected in sedentary behavior, which does not require much time to change. The increase in leisure time after retirement is immediately accompanied by an increase in sedentary behavior. The lag effect refers to the fact that residents' health behaviors begin to change significantly 2–3 years after retirement, which is mainly reflected in smoking and exercise behaviors. From the perspective of healthy behaviors, it takes time to change health behaviors, especially addictive behaviors such as smoking. A study based on French data found that women had decreased odds of smoking after 5 years compared with 1 year since retirement (26). From the perspective of the retirement system, the transition process of retirement can be divided into three stages, and healthy behavior can be changed after the transition to the stable period of retirement (24).

Evaluating the association between retirement and health behavior is useful in policies aimed at the achievement of healthy aging. Smoking, drinking, and exercise are the most important indicators of a healthy lifestyle and are associated with many health outcomes in midlife and later life (62, 63). The results showed that the realization of healthy aging by improving healthy behaviors could be attained during this period. First, the delayed retirement age system should fully consider the positive impact of retirement on residents' health behavior. Second, a flexible retirement system should be established. While promoting the realization of a healthy lifestyle, attention should also be paid to women and those with lower education levels. Finally, the government should clarify the delayed retirement system as soon as possible and build a good retirement environment for the public to make full use of the effect of retirement.

Nevertheless, the results should be interpreted cautiously due to several limitations. First, as previously mentioned, unbalance panel data was used in this study. Some respondents even were observed only once, which may lead to endogeneity problems and inconsistent estimators. Although the sensitivity and robustness tests were conducted, there may still be biased estimate. Second, previous studies have shown that the relationship between retirement and health behaviors is affected by an individual's attitude toward retirement (voluntary vs. involuntary) and their occupation (physical vs. mental), but this study was not able to discuss this issue due to data limitations (12). In addition, in this study, behaviors, such as smoking, drinking, and exercising, are self-reported by the respondents, and measuring error may occur due to biased memory and false reporting. This potential error is not conducive to the evaluation of retirement effects. Finally, it is important to note that the association between retirement and health behaviors found in the current study may not be generalizable to other countries with different retirement systems.

## Conclusions

Based on the China statutory retirement age, this study empirically explored the association between retirement and health behaviors. We observed that the transition to retirement was associated with a healthier lifestyle based on more exercise and reduced smoking and drinking; while worse sedentary behavior was noted. However, some gender differences in the association between retirement and health behaviors are noted, and men are more likely to be impacted by retirement. Further analysis of education heterogeneity found that those with higher education levels benefit more from retirement. Finally, we estimate the anticipatory effect, the immediate effect and the lag effect of retirement. Based on the results noted above and the current rapidly aging population, we provide advice for the government and society to promote healthy aging.

## Data availability statement

Publicly available datasets were analyzed in this study. This data can be found at: https://www.cpc.unc.edu/projects/china.

## Ethics statement

Written informed consent was obtained from the individual(s) for the publication of any potentially identifiable images or data included in this article.

## Author contributions

ZYa contributed to the study design, analyzed the data, and took the lead in the manuscript writing. NX, JM, and HL advised on statistical analysis and helped in the writing of the final draft of the manuscript. ZYu designed the study and proposed amendments. All authors have revised the manuscript and approved the submitted version.

## Funding

This research is supported by the National Natural Science Foundation of China (71973154) and Layout Foundation of the Ministry of Education of China (19YJA840006).

## Conflict of interest

The authors declare that the research was conducted in the absence of any commercial or financial relationships that could be construed as a potential conflict of interest.

## Publisher's note

All claims expressed in this article are solely those of the authors and do not necessarily represent those of their affiliated organizations, or those of the publisher, the editors and the reviewers. Any product that may be evaluated in this article, or claim that may be made by its manufacturer, is not guaranteed or endorsed by the publisher.
